# Multi-source secondhand smoke exposure and dental caries in children: a cross-sectional study

**DOI:** 10.3389/froh.2026.1774444

**Published:** 2026-03-27

**Authors:** Faisal M. Dardeer, Abdalrahman M. Ainousa, Dania Bahdila, Abdulkarim Fahad Alorabi, Yehya Saeed Alzahrani, Ghalia Y. Bhadila

**Affiliations:** 1 Department of Pediatric Dentistry, Faculty of Dentistry, King Abdulaziz University, Jeddah, Saudi Arabia; 2 General Dentist, Hayat National Hospital, Riyadh, Saudi Arabia; 3 General Dentist, Andalusia Hospital, Jeddah, Saudi Arabia

**Keywords:** children, dental caries, DMFT, parents, secondhand smoking

## Abstract

**Introduction:**

Despite warnings of secondhand smoke (SHS) dangers, children are still exposed in unregulated private spaces. This study aimed to assess the association between cumulative multi-source SHS exposure and its effect on children's oral health.

**Methods:**

This cross-sectional study collected smoking patterns and SHS exposure data from parents at King Abdulaziz University Dental Hospital using an Arabic-language questionnaire. Children's oral health was assessed using decayed, missing, and filled teeth (DMFT) scores obtained from dental records. An adjusted negative binomial regression model was used to assess the association between caries score and SHS exposure pattern, either at home, in the car, or at family gatherings, as follows: no exposure, exposure from a single source, two sources, or all three sources.

**Results:**

A total of 427 parents answered the questionnaire (children's median age was 10 years, and the median DMFT/dmft index score was 10). Overall, 55.3% of participants reported living in a household with smokers. A gradual positive association was observed between SHS exposure and the DMFT score. Children exposed to SHS from one source had significantly higher mean caries scores than non-exposed children with higher scores observed among those exposed from two sources (*p* < 0.001). The highest risk was observed in children exposed to SHS at all three sources (*p* < 0.001).

**Conclusion:**

A gradual positive association was observed between cumulative multi-source SHS exposure and mean caries scores. Children exposed to SHS from multiple sources had progressively higher mean caries scores than those not exposed.

## Introduction

Exposure to secondhand smoke (SHS) has been linked to an increased risk of multiple morbidities, including cardiovascular diseases, respiratory infections, and premature death in adults and children (1) and that there is no safe threshold of SHS exposure to children, as even brief encounters can lead to harmful effects (1). Despite numerous reports warning of the dangers of SHS, children are still exposed to nicotine smoke in their homes and public places. According to a Lancet Global Health report, the global prevalence of SHS exposure among adolescents was 62.9% in one or more days in any place (2). In Saudi Arabia, the prevalence of SHS exposure on at least one day during the past week was 30.6% at home and 39.8% in public areas (2). Although SHS exposure in public and indoor spaces in Saudi Arabia has been regulated in line with Article 8 of the WHO Framework Convention on Tobacco Control treaty (3), the challenge, locally and globally, remains in the exposure in unregulated or private spaces such as households, cars, and among extended family gatherings.

Among the harms of SHS exposure is its link to dental caries among children (4–6), one of the most prevalent childhood diseases (7). Previous reports have found a positive association between parental smoking habits and dental caries experience among children (4–6). Subsequent studies have reported that children living with smokers had an increased risk of developing dental caries in their primary teeth compared with children from nonsmoking households (5, 8–11). This leaves children vulnerable to the combined burden of unregulated SHS exposure and dental diseases, both of which can compromise their overall health and well-being.

In Saudi Arabia, the association between negative pediatric oral health outcomes and cumulative exposure to SHS in multiple spaces has yet to be explored. Hence, the objective of this study was to assess the association between cumulative multi-source SHS exposure in unregulated private spaces (homes, cars, and family gatherings) and its effect on children's oral health. This study was conducted in a single dental institution and sought to provide insights into the multi-source association between SHS exposure and dental caries rather than generating population-level estimates. This study may guide future more robust and scaled SHS assessments across dental settings and inform dental professionals to raise awareness and promote smoke-free homes and cars for better children's oral health outcomes.

## Methods

### Ethical consideration

Ethical approval was obtained from Faculty of Dentistry's Research Ethics Committee (REC-FD #013-01-22). Participants were informed that participation was voluntary and that there would be no consequences for refusing to participate or withdrawing from the study. Informed consents were obtained prior to completing the electronic survey. Participants were recruited from January 2022 to April 2022. This study was reported in accordance with the Strengthening the Reporting of Observational Studies in Epidemiology (STROBE) checklist (12).

### Design, setting, and sampling

his cross-sectional study used a convenience sampling approach. This study was conducted at the University Dental Hospital of King Abdulaziz University in Jeddah city, Saudi Arabia and recruited parents who visited the pediatric dental clinics for their children. The sample size was calculated using OpenEpi Version 3.01 (13) and based on the 95% confidence level, 5% margin of error, and 50% anticipated frequency for the maximum sample size; therefore, the required sample was 384 participants.

### Survey validity and reliability

The questionnaire was developed and administered to the parents in Arabic using Google Forms (Alphabet Inc., Mountain View, CA, USA). Responses were secured using a password-protected institutional Google account. The questionnaire was completely anonymous and did not collect information regarding name, email, or any identifiable information. The survey consisted of 34 questions, including skip logic questions. Content validity was assessed by a panel of three faculty members who assessed the questions relevance, clarity, simplicity, ambiguity, and importance. The overall mean content validity for the item-level index mean was 0.990 (±SD0.03), indicating excellent validity. Meanwhile, the test-retest reliability of the survey was tested by piloting the survey with 20 parents, of which 12 answered the retest survey after a 2-week period. All questionnaire items were assessed using Cohen's kappa (*κ*). Items *κ* ranged from (0.71-1) indicating good to excellent reliability. Two items demonstrated lower reliability scores: duration of smoking in years (*κ* = 0.47) and frequency of child's sugar intake (*κ* = 0.28). The smoking duration item was retained for the descriptive analysis of the parents smoking habits only (Table 2), while the sugar intake item was excluded from all analyses to avoid potential measurement bias. All remaining items (*n* = 32) were included in the final analyses.

### Measures

The survey collected data on the following variables:

1. **Sociodemographic variables:** respondents’ relationship to the child (mother, father, or other caregiver), marital status, employment status, nationality (Saudi or non-Saudi), parents age and educational level, number of children in the family, child gender (boy or girl), and the child's age. Family monthly income was categorized based on the most recent Household Income and Expenditure Survey available at the time of questionnaire development (14). This included the minimum wage, national average household income, and region-specific household income estimates in Makkah Region, in which Jeddah city is located.
2. **Child's oral health:** dental visit frequency, brushing frequency, and who brushes for the child at home. Overall caries experience was assessed using the decayed, missing, and filled teeth score (DMFT for permanent teeth and dmft for primary teeth). These data were extracted from the child's dental records (dental chart interface), which are routinely generated and updated during clinical examinations by the child's dental provider at the university pediatric dental clinics. Caries assessments were based on clinical assessment and dental radiographs, and subsequent review and approval by pediatric dental faculty as part of the university's standard diagnostic practice.
3. **Parents smoking habits:** method of smoking [Conventional Cigarettes, Waterpipe, Vape (Electronic Nicotine Delivery Systems; ENDS), cigars, or chew], smoking duration (<5 years, 5-10 years, or >10 years; following categorization described in previous literature (9), and the usual location of smoking (inside the house, outside the house, or in both).
4. **Second-hand smoke (SHS) exposure:**
  **Presence of a smoker parent in the household:** Assessed using the question “Who is the smoker parent in the household?” If the answers were “Both parents, father only and mother only” the child was living in a smoker household.
  **SHS exposure at home:** defined if both or either parents are: 1- smoking inside the house, or 2- in both inside and outside the house.
  **SHS exposure in the car:** Assessed using an affirmative response (Yes) to the question of whether the parent smokes in the car in the presence of the child.
  **SHS exposure in extended family gatherings:** SHS exposure in extended family gatherings: Assessed using an affirmative response (Yes) to the question: "Does another person in the house smoke near your child? (e.g., grandfather, grandmother, uncle, aunt, etc.)?”
  **SHS exposure pattern variable:** the pattern of SHS exposure at different settings was categorized as follows: no exposure, home only, car only, extended family gathering only, home and car, home and extended family gathering, car and extended family gathering, and all three settings. Patterns were then categorized into four categories (no exposure, exposure from a one source, exposure from two sources, or exposure from all three sources).

Exposure to SHS in the home, car, and family gatherings represents common private, unregulated environments associated with exposure in children. These settings also facilitate more reliable parental reporting of smoking behaviors and child exposure.

### Statistical analysis

Descriptive statistics presented summaries of the sample sociodemographic characteristics, children's oral health status and habits, and household smoking behavior patterns. Categorical variables were presented as frequencies and percentages. Normality of continuous variables (age and caries scores) was assessed using the Shapiro–Wilk test. Both variables were non-normally distributed (*p* < 0.05) and were summarized as medians with their associated 25th and 75th interquartile ranges (IQR). Negative binomial regression analysis was used for multivariable analysis because the outcome (DMFT/dmft score) were count data exhibiting over-dispersed distribution (mean=9.8, variance was 20.6 exceeding the mean, and one observation of a zero score), which violates Poisson regression assumptions. The main predictor in the first model was the presence of a smoker in the household as a binary variable. In the second model, the main predictor was the SHS exposure pattern as a categorical variable to assess the cumulative relationship between the predictor and outcome. In both models, we adjusted for nationality, child's age, family income, child dental visit pattern, and child brushing frequency. These variables represent socioeconomic status and oral health behaviors that are considered established risk and protective factors for dental caries in the literature (15, 16, 17). Despite the importance of including a child's sugar intake as a possible confounder, this variable was excluded from the analysis due to its low reliability score (*κ* = 0.28) to avoid introducing potential measurement bias. Multicollinearity were assessed using pairwise correlation (all correlation coefficients (r) were below 0.3. The regression estimates were reported as adjusted mean ratios (MR) given the cross-sectional design of the study and their associated 95% confidence intervals (CI). Statistical significance was set as a *p*-value less than 0.05. All statistical analyses were performed using Stata/IC 15.1 (StataCorp LLC).

## Results

The study included 427 participants (50.1% mothers and 49.9% fathers). Table 1 summarized the sample characteristics and children's oral health habits. The majority of families had more than three children (40.1%, *n* = 171), and 42.6% had a monthly family income between 12 K and less than 15 K SAR (42.6%, *n* = 182). The median age of the children was 10 years (IQR 8-11). Approximately 50.4% (*n* = 215) of the parents only take their children to the dentist when they are in pain, and 38.9% (*n* = 166) of the children brush their teeth once a day. Approximately 78.0% (*n* = 333) of the children brush their teeth by themselves. The median DMFT/dmft index score of the children was 10, with an IQR of 7–12.

**Table 1:** Participants sociodemographic characteristics and their children oral health habits (*N* = 427), King Abdulaziz Dental Hospital, Jeddah, Saudi Arabia, 2022.

Variables		N (%)
Who Answered the Survey	Mom	214 (50.1)
	Dad	213 (49.9)
Marital Status	Married	392 (91.8)
	Not Married (Divorced or Widowed)	35 (8.2)
Employment	Employed	288 (67.5)
	Housewife	97 (22.7)
	Freelancer	27 (6.3)
	Student or Not Employed	15 (3.5)
Nationality	Saudi	378 (88.5)
	Non-Saudi	49 (11.5)
Father's Age Category (Years)	20–29	21 (4.9)
	30–39	166 (38.9)
	40–49	179 (41.9)
	50 or more	61 (14.3)
Mother's Age Category (Years)	20–29	77 (18.0)
	30–39	207 (48.5)
	40–49	123 (28.8)
	50 or more	20 (4.7)
Father's Education Level	High School or Below	112 (26.2)
	More than High School	315 (73.8)
Mother's Education Level	High School or Below	192 (45.0)
	More than High School	235 (55.0)
Number of Children in the Family	1	23 (5.4)
	2	117 (27.4)
	3	116 (27.2)
	More than 3	171 (40.1)
Family Monthly Income (In Saudi Riyals; SAR)	less than 6k/month	25 (5.9)
	6k to less than 9k/month	66 (15.5)
	9k to less than 12k/month	89 (20.8)
	12 K to less than 15k/month	182 (42.6)
	More than 15 K/month	65 (15.2)
Child Gender	Boy	223 (52.2)
	Girl	204 (47.8)
Median Child's age in Years [IQR]	10 [8–11]	
Median DMFT/dmft Index Score [IQR]	Decayed	5 [2–9]
	Missing due to caries	1 [0–3]
	Filled	2 [0–4]
	Total DMFT/dmft score	10 [7–12]
In the past 12 months, how many times did your child visit the dentist?	Did not visit the dentist	35 (8.2)
	Only when in pain	215 (50.4)
	Once a year	151 (35.4)
	More than once a year	26 (6.1)
How many times does your child brush his/her teeth a day?	Less than once a day	93 (21.8)
	Once a day	166 (38.9)
	More than once a day	168 (39.3)
Who brushes for the child?	Child only	333 (78.0)
	Shared brushing (Child and Caregiver)	77 (18.0)
	Caregiver only	17 (4.0)

IQR: 25th-75th Interquartile Range.

Table 2 stratified the smoking behavior by parents. Only 2.8% (*n* = 12) reported that the mother is a smoker. Most fathers who smoke (55.0%, *n* = 235) reported conventional cigarette (CC) use (49.0%, *n* = 209), compared to no use by mothers. Approximately 18.5% of the fathers (*n* = 79) are current shisha smokers compared to 2.6% (*n* = 11) of mothers. ENDS use was reported by 8.0% of fathers and 0.2% of mothers. Approximately 73.2% (*n* = 172) of the fathers smoked for longer than 10 years, and most CC users are smoking one pack a day (50.0%, *n* = 110). Most of the fathers (59.2%, *n* = 139) reported smoking both inside and outside the house, compared to 63.6% of the mothers (*n* = 7), who reported only smoking outside the house.

**Table 2:** Parental smoking habits, King Abdulaziz Dental Hospital, Jeddah, Saudi Arabia, 2022.

Variable		Father (*n* = 235, 55.0% of the sample)	Mother (*n* = 12, 2.8% of the sample)
Type of Smoking Used	Conventional Cigarettes	209 (49.0)	0
	Waterpipe (Shisha)	79 (18.5)	11 (2.6)
	Vape (ENDS)	34 (8.0)	1 (0.2)
	Cigars or Chew	2 (0.2)	0
Smoking Duration	Less than 5 years	10 (4.3)	0
	Bet 5 to 10 years	53 (22.6)	6 (54.6)
	More than 10 years	172 (73.2)	5 (45.5)
Where do you usually smoke	Inside the house	8 (3.4)	1 (9.1)
	Outside the house	88 (37.5)	7 (63.6)
	Both	139 (59.2)	3 (27.3)

Table 3 demonstrated the patterns and sources of children's SHS exposure. Overall, 55.3% (*n* = 236) of the participants reported living in a smoker household. At home, 148 (34.7%) reported the presence of the child next to either parent while smoking, and 21.6% (*n* = 92) reported that another person in the house smokes near their child, such as grandparents or uncles. Additionally, 38.2% (*n* = 163) reported that the child was present while either parent was smoking in the car. To summarize the combined SHS exposure experience of the children, we analyzed the exposure by location and found that 18.7% (*n* = 80) were exposed to SHS at home and in the car, and (10.5%, *n* = 45) were exposed to SHS at home, in the car, and in social family gatherings. The majority of exposed children (23.4%, *n* = 100) were exposed to SHS from two sources: home, car, or family gatherings.

**Table 3:** Secondhand smoking exposure characteristics (*N* = 427), King Abdulaziz Dental Hospital, Jeddah, Saudi Arabia, 2022.

Variables		N (%)
Presence of a smoker parent in the house (Either one or both are smokers)	Yes	236 (55.3)
	No	191 (44.7)
SHS exposure at home (If either parent smokes inside the house)	Yes	148 (34.7)
	No	279 (65.3)
SHS exposure in the car (If either parent smokes inside the car in the presence of the child)	Yes	163 (38.2)
	No	264 (61.8)
SHS exposure in family gatherings (If another person smokes near the child (e.g., grandparent, uncle, aunt, etc.)	Yes	92 (21.6)
	No	335 (78.5)
Combined Patterns and Sources of SHS Exposure in Private and Unregulated Spaces		
No SHS exposure		214 (50.1)
Exposure from one source		68 (15.9)
Home only		20 (4.7)^a^
Car only		21 (4.9)^a^
Family gatherings only		27 (6.3)^a^
Exposure from two sources		100 (23.4)
Home and car		80 (18.7)^a^
Home and family gatherings		3 (0.7)^a^
Car and family gatherings		17 (4.0)^a^
Exposure from all three (home, car, and family gatherings)		45 (10.5)

^a^ The percentage is out of the overall participants (Denominator: *N* = 427, 100%).

Table 4 shows the multivariable negative binomial regression models for the association between DMFT/dmft as the outcome and the presence of a smoking parent in the household, adjusted for nationality, age, family income, child dental visit pattern, and child brushing frequency. On average, and after adjusting for the other variables, living in a smoker household significantly increased the mean caries score [MR: 1.52, 95%CI: 1.40-1.66, *p* < 0.001] compared with children living in a non-smoker household.

**Table 4:** Multivariable negative binomial regression for the association between the child's dental caries experience and the presence of a smoker parent in the household (*N* = 427), King Abdulaziz Dental Hospital, Jeddah, Saudi Arabia, 2022.

Predictors	Outcome (DMFT/dmft Score)	
	Mean Ratio [95% Confidence Interval]	*P*-value
Presence of a smoker parent in the household		
(Ref: No smoker at home)		
Either one or both parents are smokers	1.52 [1.40–1.66]	<0.001*
Child's Age (In Years)	0.99 [0.98–1.02]	0.742
Nationality (Ref: Saudi)		
Non-Saudi	1.29 [1.12–1.49]	<0.001*
Family Monthly Income in Descending Order (Ref: >15 K Saudi Riyals)	1.01 [0.98–1.06]	0.516
Frequency of Child's Dental Visits (Ref: Once a Year)		
Did not visit the dentist before	1.37 [1.16–1.61]	<0.001*
Only when in pain	1.18 [1.08–1.29]	<0.001*
Two or more times a year	1.01 [0.80–1.29]	0.922
Child's Brushing Frequency (Ref: At least Once a Day)		
Less than once a day	1.04 [0.95–1.13]	0.396
More than once a day	0.92 [0.85–1.01]	0.077
Constant	6.74 [5.41–8.41]	<0.001*

*Statistical significance (*p*-value less than 0.05).

Meanwhile, Table 5 showed the adjusted association between the DMFT/dmft scores as the outcome and the patterns of SHS exposure. A clear, gradual, positive association was observed between SHS and mean DMFT. Children exposed to SHS from one source (either at home, in the car, or at family gatherings) had higher mean DMFT/dmft scores than non-exposed children [MR: 1.26 (95%CI:1.14-1.39), *p* < 0.001]. Meanwhile, children exposed to SHS from two sources had higher mean DMFT/dmft scores than non-exposed children [MR: 1.41 (95%CI: 1.30-1.54), *p* < 0.001]. Finally, the highest risk was observed in children exposed to SHS at home, in cars, and at extended family gatherings [MR: 1.78 (95%CI: 1.60-1.97), *p* < 0.001].

**Table 5:** Multivariable negative binomial regression for the association between the child's dental caries experience and patterns of secondhand exposure (*N* = 427), King Abdulaziz Dental Hospital, Jeddah, Saudi Arabia, 2022.

Predictors	Outcome (DMFT/dmft Score)	
	Mean Ratio [95% Confidence Interval]	*P*-value
Number of SHS Exposure Sources (Ref: No SHS Exposure) Sources: Home, Car or Family Gathering		
Single Source	1.26 [1.14–1.39]	<0.001*
Two Sources	1.41 [1.30–1.54]	<0.001*
All Three Sources	1.78 [1.60–1.97]	<0.001*
Child's Age (In Years)	0.99 [0.97–1.01]	0.435
Nationality (Ref: Saudi)		
Non-Saudi	1.34 [1.17–1.53]	<0.001*
Family Monthly Income in Descending Order (Ref: >15 K Saudi Riyals)	1.01 [0.97–1.06]	0.620
Frequency of Dental Visits (Ref: Once a Year)		
Did not visit the dentist before	1.49 [1.25–1.77]	<0.001*
Only when in pain	1.22 [1.12–1.32]	<0.001*
Two or more times a year	1.07 [0.81–1.42]	0.648
Child's Brushing Frequency (Ref: At least Once a Day)		
Less than once a day	1.02 [0.94–1.11]	0.695
More than once a day	0.91 [0.84–1.00]	0.044
Constant	7.31 [5.91–9.04]	<0.001*

*Statistical significance (*p*-value less than 0.05).

## Discussion

The findings of this study revealed that over half of the participants lived in a household with smokers. There was a gradual positive association between cumulative multi-source SHS exposure and the mean DMFT/dmft score. Children exposed to SHS from one or two sources had progressively higher mean DMFT/dmft scores than non-exposed children. The highest risk pattern was for children who were simultaneously exposed to SHS at home, in cars, and at extended family gatherings.

SHS increases the risk of dental caries in children through a combination of direct biological effects on teeth and saliva and indirect behavioral changes in feeding styles and oral hygiene (18, 19). Regarding biological mechanisms, parental and early postnatal exposure to tobacco smoke is associated with enamel hypomineralization, which makes teeth more susceptible to caries. Nicotine and other toxicants can disturb ameloblast function, calcium/phosphate transport, and enamel maturation, thereby reducing hardness and increasing porosity (19–21). Additionally, children exposed to SHS show altered salivary flow, buffering capacity, and composition, with possible increases in the levels of inflammatory markers. Reduced salivary cleansing and buffering enhance demineralization around plaque biofilms, favoring caries development (19). Increased plaque accumulation and gingival inflammation have also been documented in SHS-exposed- schoolchildren, consistent with a smoke-modified- biofilm (4, 20). SHS weakens local immune responses (e.g., neutrophil function and secretory IgA activity), reducing host defense against cariogenic bacteria (19). Regarding behavioral factors, parental smoking correlates with less health-conscious- feeding styles and higher child BMI, both linked to increased caries risk (22, 23). Smoking by caregivers is associated with overall lower health literacy and less stress on preventive behaviors, which can lead to inadequate caries preventive- practices at home (19).

In this study, mothers reported a substantially lower prevalence of smoking than fathers. This finding is consistent with global and local studies where the prevalence of women smoking is underreported due to possible gender-based stigma, especially in conservative communities (24, 25). The discrepancy between the prevalence of maternal and paternal smoking status might not reflect the true extent of SHS exposure of the children. Therefore, although statistically significant, the reported positive association between childhood dental caries and SHS exposure in the household is likely to be conservative and underestimated. Notably, maternal smoking is consistently associated with worse health outcomes in children. A large cohort study in Scotland found that children of mothers who smoke were at a higher risk of hospitalization, acute respiratory infections, asthma, and bacterial meningitis within the first five years of life (26). A Meta-analysis has also reported that maternal smoking status is positively associated with attention-deficit/hyperactivity disorder (ADHD) in children (27). Although underreported in multiple studies, these findings highlight the importance of raising awareness about the effects of parental smoking on children's health outcomes, particularly maternal smoking, given that mothers are most often the first-line caregivers.

Furthermore, we identified a gradual positive association between SHS exposure in multiple locations and mean DMFT/dmft scores. Children exposed to SHS from one or two sources had progressively higher mean DMFT/dmft scores than non-exposed children. The highest risk pattern was for children who were simultaneously exposed to SHS at home, in cars, and at extended family gatherings. We also found that most fathers in our sample reported being smokers for more than 10 years, smoking both inside and outside the house. This finding indicates frequent, prolonged, and multi-source unregulated exposure to SHS among children. In households, a study conducted in multi-unit housing in Singapore found that residents who were exposed to SHS from within the home or even from neighboring units were exposed to higher indoor PM2.5 levels, a standard biomarker for quantifying SHS exposure (28). This was also associated with worse self-reported respiratory health (28). In cars, active exposure to SHS is detrimental as reported by multiple air monitoring studies, where SHS biomarkers are mostly revealed among children and adolescents (29). Children are more vulnerable to SHS exposure within the family car due to their smaller body mass and higher ventilation rates to adults. Therefore, they inhale higher doses of SHS for the same level of exposure (30).

In a mission to regulate SHS exposure in cars, an evaluation of the legislation implementing a smoke-free vehicle in England, Scotland and Wales found a 22% relative reduction in exposure among children (31). However, the debate remains about the complex intersection of adults’ rights to privacy and children's rights to thrive in a smoke-free environment. Another underrecognized source of SHS exposure for children is family gatherings, which are frequent and an important pillar of the Saudi family. Over one fifth of our sample reported that another person in the house smokes near their child such as grandparents or uncles. Depending on the type of gathering, these exposures can be prolonged and involve multiple active smokers. Despite awareness of the potential harms of SHS exposure, caregivers may be hesitant to restrict smoking by elder family members or guests. In multi-smokers’ settings, a study across 11 European countries found that the concentrations of airborne nicotine, which is used as a marker of SHS, were positively associated with enclosed venues, the presence of more than two smokers, and the number of discarded cigarette butts (32). These findings highlight the possible implications of the social and familial contexts in which SHS exposure occurs.

This study had some limitations. First, it was conducted in a single dental institution to drive insights into the cumulative multi-source association of SHS exposure in private, unregulated spaces and dental caries rather than generating population-level estimates, and therefore the findings are not representative of the general pediatric population. Second, the survey was self-reported and subject to recall and desirability bias, which might risk misclassification. Third, the cross-sectional design of this study cannot support temporal or causal relationships between SHS exposure and dental caries, as both variables were assessed concurrently. Fourth, the median age of the study sample was 10 years, which reflects the natural age distribution of children attending pediatric dental clinics in this population rather than an intentional focus on mixed dentition. In this study population, children tended to visit the dentist at a relatively older age, which might have limited the representation of younger children in the current findings. Previous studies in this population have reported that the mean age of the first dental visit was approximately 6 years (±SD 2.1) (33). Finally, this study used a combined DMFT/dmft to measure children's overall caries experience. While this approach captured the overall caries burden, it may have introduced measurement heterogeneity and limited direct comparability with studies reporting DMFT and dmft separately. Despite its limitations, this study may guide future more robust and scaled SHS assessments across dental settings and inform dental professionals to raise awareness and promote smoke-free homes and cars for better children's oral health outcomes. Future studies should assess the biological and behavioral mechanisms by which SHS exposure is associated with the severity of dental caries in children. Additionally, exploring the social and familial contexts in which SHS exposure occurs can guide culturally informed community advocacy and control.

In conclusion, there was a gradual positive association between cumulative multi-source SHS exposure and the mean DMFT/dmft score. Children exposed to SHS from one or two sources had progressively higher mean DMFT/dmft scores than non-exposed children. The highest risk pattern was for children who were simultaneously exposed to SHS at home, in cars, and at extended family gatherings. These findings may inform dental professionals to raise awareness and promote smoke-free homes and cars for better oral health outcomes in children.

## Data Availability

The original contributions presented in the study are included in the article/Supplementary Material, further inquiries can be directed to the corresponding author.
